# Soluble CD163 is a predictor of fibrosis and hepatocellular carcinoma development in nonalcoholic steatohepatitis

**DOI:** 10.1186/s12876-023-02786-4

**Published:** 2023-05-10

**Authors:** Miwa Kawanaka, Ken Nishino, Mayuko Kawada, Katsunori Ishii, Tomohiro Tanikawa, Ryo Katsumata, Noriyo Urata, Jun Nakamura, Mitsuhiko Suehiro, Ken Haruma, Hirofumi Kawamoto

**Affiliations:** Department of General Internal Medicine, General Medical Center, Kawasaki Medical School, 2-6-1, Nakasange, Kita-ku, Okayama City, 700-8505 Okayama Japan

**Keywords:** Nonalcoholic fatty liver disease, Soluble CD163, Hepatocellular carcinoma, Cirrhosis, Prognosis

## Abstract

**Background:**

Nonalcoholic fatty liver disease (NAFLD) is the most common cause of chronic liver disease. The serum level of soluble CD163 (sCD163), a macrophage activation marker, is associated with liver tissue changes; however, its prognostic value is unknown. Here, we determined the utility of sCD163 as a marker for hepatocellular carcinoma (HCC) and prognostic marker for NAFLD.

**Methods:**

This retrospective study obtained data regarding serum sCD163 levels, liver histology, and background factors associated with NAFLD in 287 patients (men/women, 140/147; average age, 53 ± 14 years) with NAFLD who underwent liver biopsy. Repeated liver biopsies of 287 patients with NAFLD (5.0 ± 2.7 years) were compared regarding serum sCD163 levels and liver tissue changes (stage, grade, steatosis, and NAFLD activity score).

**Results:**

Serum sCD163 levels increased with the progression of liver fibrosis and inflammation (both P < 0.05) and were particularly helpful in distinguishing cases of Grade 4 fibrosis (P < 0.001). Levels of sCD163 significantly decreased in patients with NAFLD exhibiting alleviated fibrosis and inflammation (P < 0.05). We could also predict the development of HCC and associated mortality based on serum sCD163 levels at the time of NAFLD diagnosis. Serum sCD163 levels were higher in patients with HCC than in patients without HCC (1074 ± 379 ng/ml vs. 669 ± 261 ng/ml; P < 0.0001), and the same trend was observed for mortality.

**Conclusions:**

The serum sCD163 level reflects the progression of fibrosis and inflammation in liver tissues, showing much promise as a noninvasive biomarker for nonalcoholic steatohepatitis and NAFLD as well as a possible predictor of HCC development and patient prognosis.

**Supplementary Information:**

The online version contains supplementary material available at 10.1186/s12876-023-02786-4.

## Background

Nonalcoholic fatty liver disease (NAFLD) is the most common cause of chronic liver disease, affecting approximately 25% of the general adult population worldwide [1]. The major causes of death in patients with NAFLD are cardiovascular disease, liver-related diseases including hepatocellular carcinoma (HCC), and other malignancies [2, 3]. NAFLD is the leading cause of HCC in some countries, and the number of patients with HCC due to NAFLD is rapidly increasing [4, 5].

It is generally known that the mortality rate of patients with nonalcoholic steatohepatitis (NASH) is high [1]. Among these patients, the prognosis of patients with NAFLD and advanced hepatic fibrosis is poor [1, 6–11]; therefore, identifying biomarkers that can predict the prognosis and risk of HCC in patients with NASH is crucial. The pathogenesis of NASH/NAFLD has been linked to pathogen-associated molecular patterns (e.g., gut microbiota) that enter from the intestinal tract and affect hepatic macrophages [12]. Macrophages play an important role in the development of inflammation and fibrosis in the liver. CD163 is a scavenger receptor for surface hemoglobin and haptoglobin and is expressed on macrophages and monocytes [13]. Upon macrophage activation, its expression is increased and it is released into the bloodstream as soluble CD163 (sCD163) by inflammatory signals containing lipopolysaccharide (LPS) [13, 14]. Additionally, sCD163 is associated with insulin resistance and lipolysis [12, 15]. Despite reports that serum sCD163 levels in NAFLD are associated with changes in liver tissue, there are no studies evaluating the same. Therefore, in this study, we investigated the relationship between serum sCD163 levels and histological changes, liver carcinogenesis, and prognosis of liver disease.

## Methods

We retrospectively identified 287 patients with NAFLD who underwent liver biopsy at the Kawasaki Medical School General Medical Center between January 1996 and December 2018. The exclusion criteria were as follows: a history of other liver diseases (including hepatitis B or hepatitis C virus infections, autoimmune liver diseases, drug-induced liver injury, and metabolic liver diseases) and a history of high alcohol intake (men: ≥30 g/day; women: ≥20 g/day). A blood test was performed prior to liver biopsy to determine the association between sCD163 levels and liver fibrosis (grade, steatosis, ballooning, NAFLD activity score [NAS]) [16, 17]. Serum sCD163 levels in 119 patients with NAFLD who underwent repeated liver biopsies were assessed, and changes in liver histology were examined. The treatment of 119 patients with NAFLD who underwent repeated biopsies was based on diet and exercise recommendations and treatment of lifestyle-related diseases, but not the use of therapeutic agents for NAFLD. We subsequently investigated whether sCD163 levels at the time of NAFLD diagnosis could predict the carcinogenesis and prognosis of HCC in 243 patients who were followed up.

The sCD163 levels were assessed using an enzyme-linked immunosorbent assay (ELISA) kit (CD163, Human ELISA Kit Quantikaine; R&D Systems Inc., Minneapolis, MN, USA) and the primary antibody used was a mouse monoclonal antibody. Human CD163 (10D6; Leica Biosystems, Milton Keynes, UK) was diluted 1:50 prior to the analyses. All experimental protocols were approved by the Research Ethics Committee of Kawasaki Medical School (No. 2088) and complied with the guidelines of the 1975 Helsinki Declaration. Each patient provided informed consent signed at the time of histological diagnosis of the liver and opted out on the Internet.

### Liver biopsy and histological analysis

All liver biopsies were ultrasound-guided and performed using 16G or 17G biopsy needles or laparoscopy-guided using 14G needles. The liver core tissue collected was 2.5 cm long and the number of portal veins was ≥ 5. Histological examinations were performed by two experienced liver pathologists who were blinded to the patient details. The histological parameters included fibrosis, inflammation, steatosis, hepatocyte ballooning, and the NAS system [16]. The individual histological features of NAFLD were assessed using the following NAS system proposed by the NASH Clinical Research Network (NASH CRN): lobular inflammation (0–3), steatosis (0–3), and hepatocellular ballooning (0–2). The liver fibrosis stages were assessed according to Brunt’s criteria [17]. Fibrosis progression was defined as progression of at least 1 stage, improvement was defined as improvement of 1 stage, and static status was defined according to Brunt’s criteria [17]. Inflammation grade was defined as progression of at least 1 stage, improvement was defined as improvement of 1 stage, and static status was defined using the following NAS system [16].

### Statistical analysis

Cumulative all-cause mortality and HCC carcinogenic events during follow-up were assessed using the Kaplan–Meier method, and compared using the log-rank test. Kaplan–Meier analysis included variations in serum sCD163 levels. We also calculated odds ratios and performed receiver operating characteristic (ROC) curve analysis using logistic regression analysis to determine the best predictive cutoff value for serum sCD163 values. The optimal cutoff value was determined based on the Youden index. The prognostic performance of the optimal cutoff value was expressed as diagnostic specificity, sensitivity, positive predictive value (PPV), and negative predictive value (NPV) by analyzing the area under the ROC curve (AUC). The normality of the distribution of continuous variables was evaluated using the Kolmogorov–Smirnov test.

The Wilcoxson test was used to assess the stage and grade of liver carcinoma, serum sCD163 levels, as well as the influence of background factors on repeated liver biopsies and changes in blood test results, serum sCD163 levels, and liver tissue. P < 0.05 was considered significant, and all statistical analyses were performed using JMP software (version 14.2; SAS Institute Inc., Carey, NC, USA).

## Results

### Relationship between sCD163 and liver histology

**Fig. 1:**
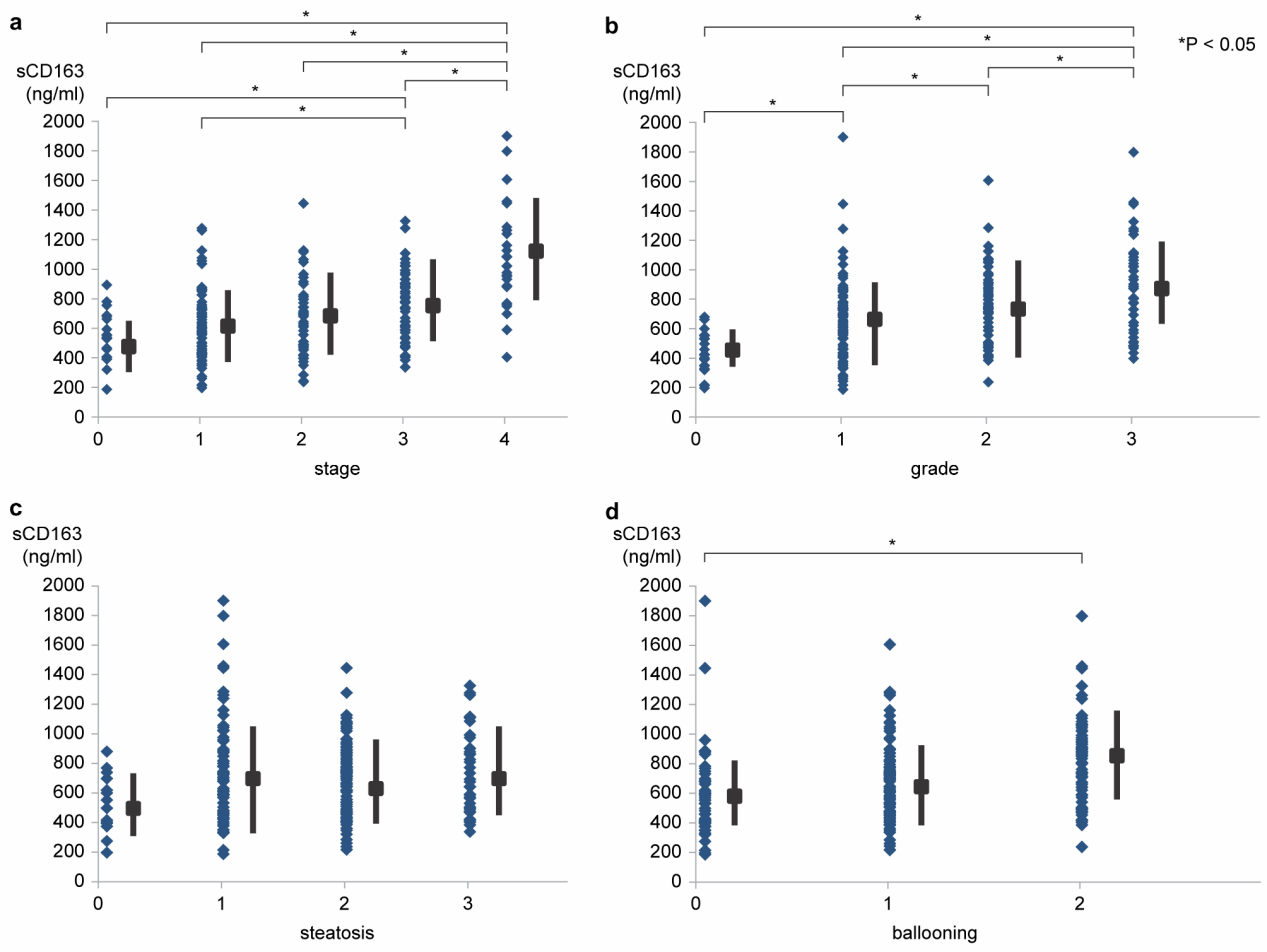
Relationship between sCD163 and liver histology (**a**). Stage (**b**). Grade (**c**). Steatosis (**d**). Ballooning The serum sCD163 levels significantly increased with the progression of fibrosis, and correlated with inflammation grade and hepatocyte ballooning (Fig. 1a, b, d and P < 0.05). However, serum sCD163 levels were not associated with steatosis (Fig. 1c; P = N.S). sCD163, soluble CD163

In this study, serum sCD163 levels were measured in 287 patients with NAFLD who underwent liver biopsy at baseline (Table 1). The serum sCD163 levels significantly increased with the progression of fibrosis and were correlated with both inflammation grade and hepatocyte ballooning (P < 0.05); however, they were not related to steatosis (Fig. 1a–d). The serum sCD163 levels were useful for the diagnosis of fibrosis stage 3–4, especially stage 4 (AUC, 0.85; cutoff, 932 ng/ml; sensitivity, 72.0%; specificity, 60.2%; PPV, 40.9%; NPV, 96.5%). The serum sCD163 levels were high in Grade 2–3 (AUC, 0.70; cutoff, 761 ng/ml; sensitivity, 52.9%; specificity, 34.4%; PPV, 70.4%; NPV, 65.6%) and NAS ≥ 5(AUC, 0.65; cutoff, 761 ng/ml; sensitivity, 50%; specificity, 28.9%; PPV, 68.6%; NPV, 63.1%) (Figure S1, a–d). The serum sCD163 was also able to distinguish between NASH and simple steatosis. We investigated whether sCD163 level is an independent marker for diagnosing NAFLD and advanced fibrosis (≥ stage3) by multivariate analysis. Univariate and multivariate analysis showed that sCD163 level is an independent marker for diagnosing progressive fibrosis in NAFLD (Table 2).

**Table 1:** Clinical and histological characteristics of patients at baseline

	n = 287
Age (years)	53 ± 14
Male/Female	140/147
BMI (kg/m^2^)	27.6 ± 3.7
Fibrosis, 0/1/2/3/4	37/88/64/74/24
Grade, 0/1/2/3	21/134/91/41
Steatosis, 0/1/2/3	0/1/2/3
NAS score, < 4/≧5	149/138
ALT(IU/l)	56 (10–281)
AST(IU/l)	37 (13–198)
γ-GTP (IU/l)	49 (11–560)
Total Bilirubin (mg/dl)	0.7 (0.2–1.7)
Total Cholesterol (ng/dl)	198 (102–317)
Cholinesterase (IU/l)	268 (71–526)
Platelet count (10^4^/ug)	19.4 (7.9–43.3)
HOMA-IR	2.9 (0.7–19.8)
Iron (ug/dl)	118 (31–261)
Ferritin (ng/dl)	125 (1.7–1188)
Leptin (ng/dl)	10.3 (1.1–54.6)
Adiponectin (ug/ml)	5.5 (2.0–27.5)
high sensivity-CRP (mg/dl)	0.117 (0.01–1.92)
P-III-P (U/ml)	0.7 (0.15–1.9)
Type 4 collagen7S (ng/ml)	4 (2.5–15)
Hyaluronic acid (ng/ml)	36 (9–521)
TIMP-1(ng/ml)	172.4 (94–432)
WFA^+^M2BP (C.O.I)	0.97 (0.21–18.2)
sCD163(ng/ml)	676 (186–1900)

BMI, Body mass index; ALT, alanine aminotransferase; AST, aspartate aminotransferase; γ-GTP, γ-glutamyltransferase; HOMA-IR, homeostasis model assessment-insulin resistance level; P-III-P, procollagen-III-peptide; TIMP-1, Tissue inhibitor of metalloproteinases-1; WFA^+^M2BP, Wisteria floribunda agglutinin Mac-2 binding protein; sCD163, soluble CD163.

**Table 2:** Factors of advance fibrosis progression in NAFLD patients in univariate and multivariate analysis

	Univariate analysis			Multivariate analysis		
	AUC	CI	P-value	Odds ratio	95%CI	P-value
Age	0.63	1.03–9.23	0.003	5.6	1.12–30.1	0.0356
Gender (F)	0.61	0.36–1.04	0.0707	0.72	0.35–1.43	0.3472
ALT	0.4146		0.4146			
AST	0.656	0.58–0.72	0.0061	4.05	0.61–27.7	0.1451
Platelet count	0.6132	0.5399–0.6866	0.0199	1.74	0.22–13.2	0.5931
Ferritin	0.56	0.4783–0.6389	0.0161	2.51	0.2–34.5	0.4756
Total Cholesterol	0.767		0.767			
Alubumin	0.6448	0.5709–0.7170	0.0002	0.11	0.01–0.7	0.023
P-III-P	0.6567	0.5858–0.7285	< 0.0001			
Type 4 collagen7S	0.7699	0.7008–0.8342	< 0.0001			
Hyaluronic acid	0.7588	0.6927–0.8236	< 0.0001			
WFA^+^M2BP	0.6879	0.6121–0.7622	< 0.0001			
sCD163	0.7262	0.6551–0.7911	< 0.0001	43.9	4.86–481	0.0006

CI; confidence interval, ALT, alanine aminotransferase; AST, aspartate aminotransferase; P-III-P, procollagen-III-peptide, WFA^+^M2BP, Wisteria floribunda agglutinin Mac-2 binding protein; sCD163, soluble CD163.

### Relationship between sCD163 and liver fibrosis markers

The diagnostic ability of sCD163 for fibrosis stage 3 or higher was compared with other existing serum markers such as type 4 collagen7S, hyaluronic acid, TIMP-1, P-III-P, WFA + M2BP by AUC, sensitivity, specificity, PPV, and NPV and listed in Table S1. The diagnostic ability of sCD163 for advance fibrosis was excellent, comparable to type 4 collagen 7 S and hyaluronic acid. Furthermore, serum sCD163 showed a strong positive correlation with liver fibrosis markers, especially type 4 collagen 7 S, hyaluronic acid, and P-III-P (Fig. S2).

### Relationship between tissue changes in repeated liver biopsies and sCD163

**Fig. 2:**
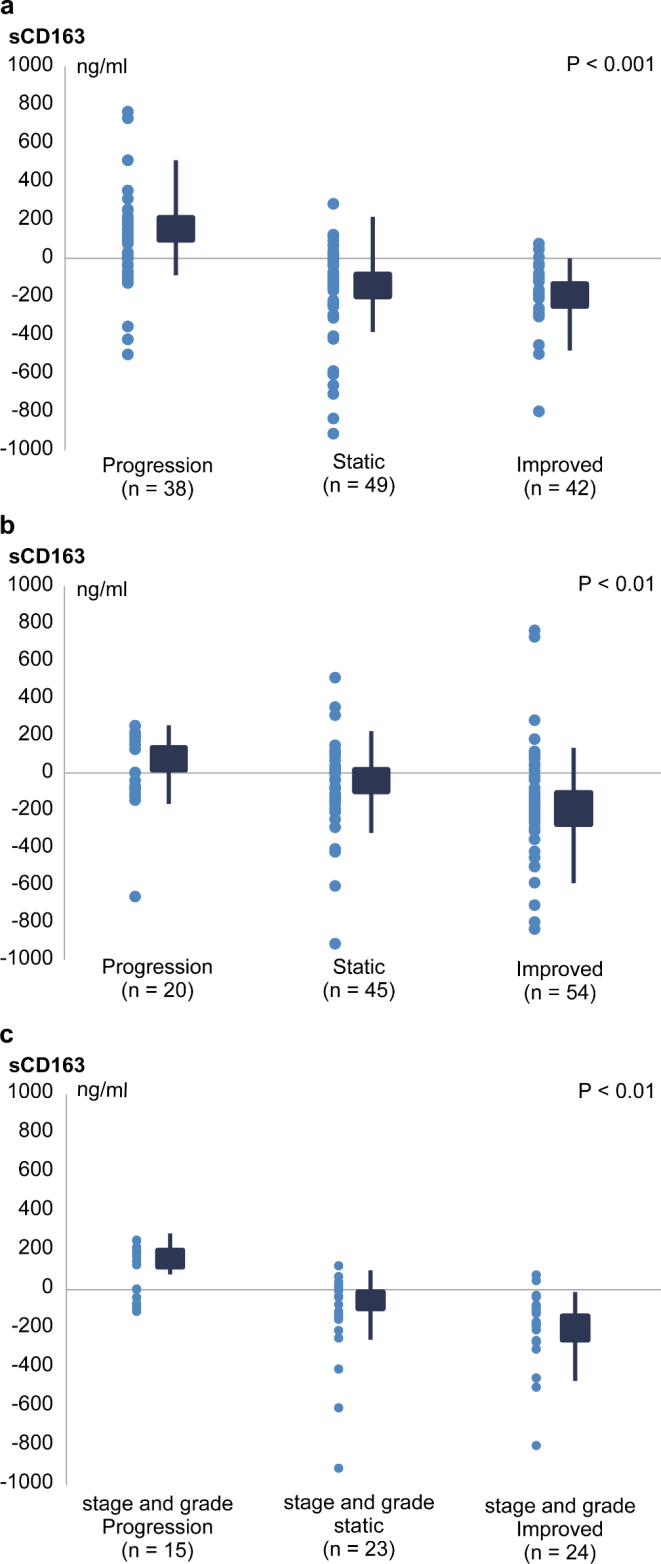
Assessment of changes in the serum sCD163 levels in patients who underwent repeated biopsies (**a**). Liver stage (**b**). Grade (**c**). Stage and grade The difference in serum sCD163 levels between the first and second liver biopsies increased as fibrosis and inflammation progressed and decreased as fibrosis and inflammation improved

Table 3 shows the clinical features and biomarker data of both biopsies for 119 patients with NAFLD who underwent repeated biopsies. Fibrosis was progressed, static, and improved in 32%, 41%, and 26% of the patients, respectively; inflammation grade was progressed, static, and improved in 18%, 37%, and 45% of the patients, respectively. Steatosis was progressed, static, and improved in 20%, 40%, and 40% of the patients, respectively (see Figure S3 in Additional file 1). The difference in levels of serum sCD163 between the first and second liver biopsies increased when both inflammation and fibrosis progressed and decreased when both fibrosis and inflammation improved (Fig. 2a–c).

**Table 3:** Patients with NAFLD who underwent repeated liver biopsies

Clinical and histological characteristics				
		n = 119		
Age, years		50.2 ± 14.2		
Male/Female		64/55		
BMI (kg/m^2^)		27.9 ± 3.6		
Fibrosis, 0/1/2/3/4		12/42/27/37/1		
Grade, 0/1/2/3		9/45/42/23		
Steatosis, 0/1/2/3		1/37/51/30		
Biomarkers in NAFLD and NASH				
	**First time**		**Second time**	**P-value**
ALT (IU/l)	91 ± 55		45 ± 34	< 0.0001
AST (IU/l)	54.2 ± 27.2		36.6 ± 19.7	< 0.0001
γ-GTP (IU/l)	77.4 ± 54.4		55.7 ± 57	< 0.0001
T- Bilirubin (mg/dl)	0.7 ± 0.3		0.8 ± 0.3	0.1124
Total Cholesterol (ng/dl)	207 ± 35.7		189 ± 35.8	0.0012
Cholinesterase (IU/l)	255 ± 98		330 ± 91.7	< 0.0001
Platelet count (10^4^/ug)	21.3 ± 6.1		20.6 ± 6.5	0.4892
HOMA-I R	3.8 ± 2.8		3.2 ± 2.0	0.1738
Iron (ug/dl)	122 ± 37.9		116 ± 37.9	0.2847
Ferritin (ng/dl)	219 ± 183		138 ± 140	0.0001
Leptin (ng/dl)	11.4 ± 6.5		12.0 ± 7.7	0.8855
Adiponectin (ug/ml)	5.9 ± 2.5		6.6 ± 3.5	0.4023
High-sensitivity CRP (mg/dl)	178 ± 153		174 ± 298	0.0012
P-III-P (U/ml)	0.7 ± 0.1		0.6 ± 0.1	< 0.0001
Type 4 collagen 7 S (ng/ml)	4.1 ± 0.8		3.9 ± 0.9	0.1841
Hyaluronic acid (ng/ml)	45.3 ± 48.2		54.1 ± 50	0.0925
TIMP-1 (ng/ml)	180 ± 42.2		156 ± 55.2	0.0234
CK18 (micro/l)	705 ± 520		566 ± 475	0.0152
M2BPGi (C.O.I)	1.05 ± 0.5		0.91 ± 0.49	0.0297
sCD163 (ng/ml)	742 ± 298		658 ± 290	0.0122

BMI, body mass index; NAFLD, nonalcoholic fatty liver disease; NASH, nonalcoholic steatohepatitis; sCD163, soluble CD163

### Relationship of sCD163 levels with NASH prognosis and liver carcinogenesis

**Fig. 3:**
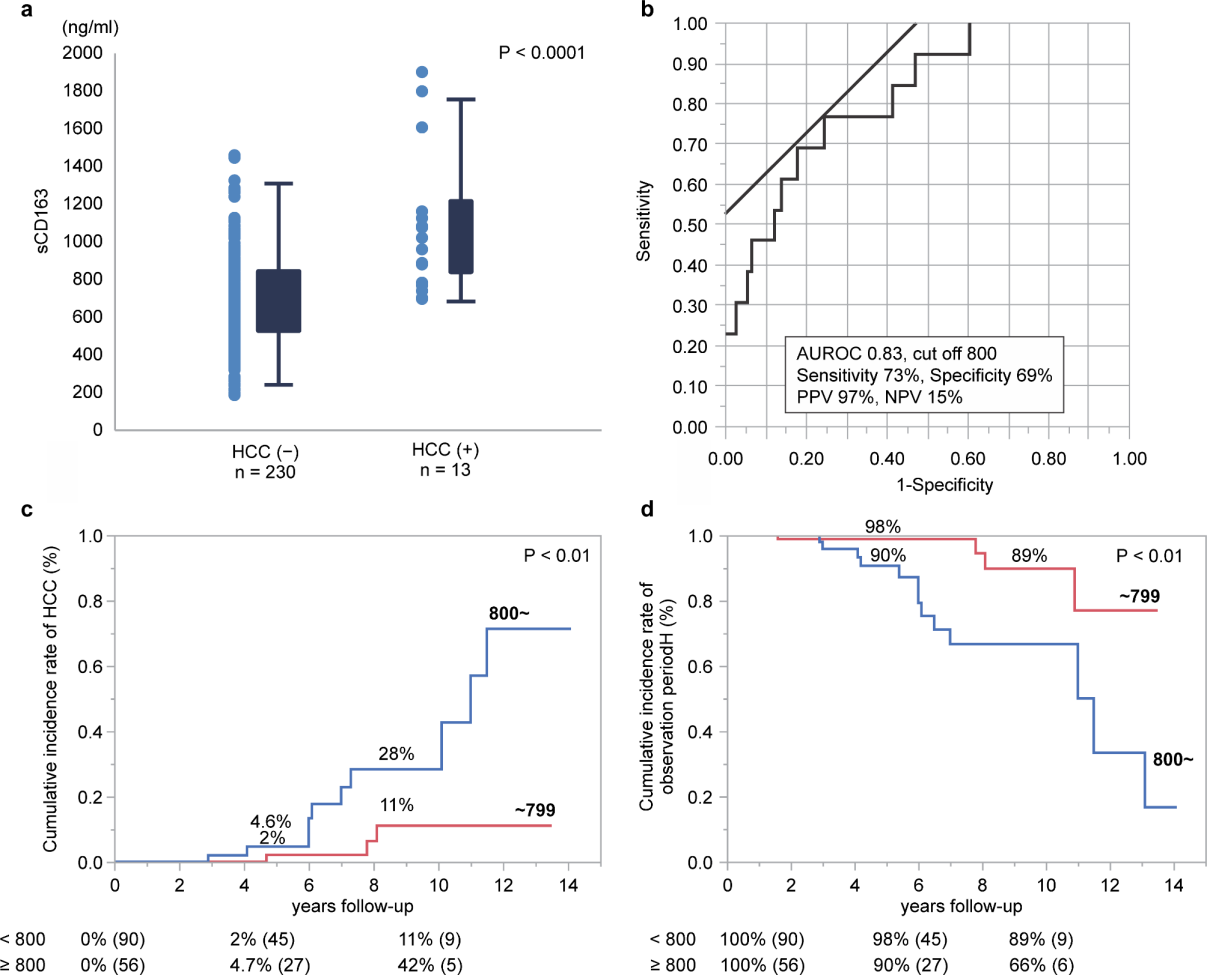
Serum sCD163 levels in NAFLD with and without HCC. (**a**) The serum sCD163 levels were significantly greater in patients with NAFLD exhibiting HCC than in those without HCC (P < 0.0001) (**b**) Predictive performance of sCD163 for HCC development in patients with HCC. The area under the receiver operating characteristic curve value for prediction of HCC development was 0.83 with sensitivity of 73% and specificity of 69% (**c**) In cases with serum sCD163 levels < 800 ng/ml, the 5- and 10-year liver carcinogenic rates were 2% and 11%, respectively. In cases with > 800 ng/ml, the 5- and 10-year liver carcinogenic rates were 4.7% and 42%, respectively (P < 0.01). (d) Cumulative survival at 5 and 10 years was 98% and 89%, respectively, when serum sCD163 levels were < 800 ng/ml; cumulative survival at 5 and 10 years was 90% and 66%, respectively, when serum sCD163 levels were > 800 ng/ml (P < 0.01) (Observation period 4.8 (1-14.1) years

Survival curves and HCC carcinogenesis curves were created using serum sCD163 levels. To investigate the predictive performance of these biomarkers for NAFLD mortality and HCC carcinogenesis, the optimal cutoff for serum sCD163 levels was determined based on ROC curve analysis of all 243 patients with NAFLD (Fig. 3a). As shown in Fig. 3b, the cutoff value for serum sCD163 levels was set at 800 ng/ml. From 243 patients with traceable sCD163 levels at the time of NAFLD diagnosis, 13 patients had HCC onset and 15 patients died (10 patients had liver-related deaths, 3 patients had cancers of other organs, and two patients had cardiovascular events). The median observation period was 4.8 (1–14.1) years.

In cases with serum sCD163 levels < 800 ng/ml, the 5- and 10-year liver carcinogenic rates were 2% and 11%, respectively. Conversely, in cases where serum sCD163 levels were ≥ 800 ng/ml, the 5- and 10-year liver carcinogenic rates were 4.7% and 42%, respectively (P < 0.01) (Fig. 3c). At the time of NASH diagnosis, the 5- and 10-year survival rates for patients with serum sCD163 levels < 800 ng/ml were 98% and 89%, respectively. Conversely, for patients with serum sCD163 levels ≥ 800 ng/ml, the 5- and 10-year survival rates were 90% and 66%, respectively. Survival was significantly reduced when serum sCD163 levels were 800 ng/ml or higher (P < 0.01) (Fig. 3d). Thus, patients with serum sCD163 levels > 800 ng/ml had a poorer prognosis than those with serum sCD163 levels < 800 ng/ml.

## Discussion

Macrophage activation is an important part of the etiology of several diseases, and serum CD163 levels are associated with obesity, sepsis, insulin resistance, diabetes, NASH, and portal hypertension [18, 22]. Additionally, liver macrophages play an important role in inflammation and fibrosis in both NAFLD and NASH [12, 19–24]. In this study, immunostaining of the macrophage marker CD68 and macrophage activation marker sCD163 was performed to determine whether blood macrophage levels were associated with liver macrophage activation. The positive staining of sCD163 (M2 macrophages), in which sinusoid and portal vein areas were observed, was consistent with that of CD68, indicating whole macrophages (see Figure S4 in Additional file 1). Among macrophages, M1 macrophages are attributable to inflammation, while M2 macrophages reflect repair.

M2 macrophages are involved in developments including liver fibrosis, suggesting that repair by macrophages occurs in the liver.

A 12-month study of children with obesity reported a strong association between the levels of serum sCD163 and liver enzymes [25]; however, there are no reports on the relationship between sCD163 levels and changes in liver tissue. This study is the first to examine the association between changes in liver tissue and sCD163 levels. Changes in sCD163 levels were associated with changes in fibrosis and inflammation in 119 patients with NAFLD who underwent repeated liver biopsies. Serum sCD163 levels increased in cases where both fibrosis and inflammation progressed and decreased where both fibrosis and inflammation improved. This suggests that the serum sCD163 level is a useful biomarker that represents changes in liver tissue. Additionally, repeated liver biopsies were performed up to 5.0 years after the initial diagnosis, which is a sufficient period of time to determine tissue changes. Therefore, we concluded that changes in serum sCD163 levels are useful biomarkers for changes in liver tissue.

Hegazy et al. [12] stated that there may be an association between macrophage activation by LPS and its surface and the upregulation of CD163 in NAFLD. LPS, a cell wall component of gram-negative bacteria that is continuously released during the death of intestinal cells, is a potent proinflammatory signaling transducer acting via the TLR4/NF-kB pathway and contributes to the production of endogenous metabolic toxins [26]. Gut flora is involved in the pathology of NASH through the release of LPS, which activates macrophages; therefore, it may also be involved in the development of NAFLD [27]. In vitro experiments have reported an association of serum sCD163 levels with changes in lipid metabolism, thereby promoting macrophage activation in the liver [15].

Rosso et al. [20] reported that biopsy-proven NAFLD serum sCD163 levels were associated with circulating free fatty acids (FFAs), lipid flux, adipose tissue IR as well as with changes in lipid metabolism such as FFA overflow and activating macrophages in the liver. Furthermore, human monocyte-derived macrophages (hMDMs) were treated with palmitic acid, and the sCD163 level in the culture medium was measured. Palmitic acid exposure increased sCD163 secretion in hMDMs. Additionally, palmitic acid levels were significantly higher in patients with NAFLD and severe fibrosis than those with mild fibrosis. Although the contribution of adipose tissue macrophages to circulating sCD163 cannot be ruled out, the combination of results suggest that circulating sCD163 primarily represents liver macrophage activation in patients with NAFLD.

Our results demonstrate that cases with high serum sCD163 levels—especially cases of NAFLD with serum sCD163 levels > 800 ng/ml—had a high incidence of HCC and a poor prognosis. This suggests that patients with advanced liver fibrosis or severe inflammation are more likely to develop HCC and have a poor prognosis. Therefore, patients with NAFLD and high serum sCD163 levels should undergo screening for HCC. According to reports, tumor-related macrophages and M2 macrophages in HCC are regulated by interleukin 10, which accelerates the progression of HCC [28]. In NASH, inflammatory cytokines produced in immune cells that are involved in steatohepatitis are also involved in HCC infiltration and metastasis [29, 30]. The association between serum sCD163 levels and the liver tissue in NASH and in HCC has not been clarified. Furthermore, in both hepatitis C virus (HCV), hepatitis B virus (HBV), and metabolic association fatty liver disease (MAFLD), sCD163 increased in association with histologic fibrosis stage [31]. However, there are no reports of sCD163 in HCC, and more studies are needed in more HCC cases.

Recently, certain biomarkers have been reported to predict the development and prognosis of HCC. For example, DKK-1, an inhibitor of the Wnt/β-catenin signaling pathway involved in embryogenesis, predicts HCC development [32]. In addition, micro RNA 142 was highly expressed in HCC and microRNAs 191, 22, and 126 were higher in controls [33]. Furthermore, high sPD-L1 levels could be a possible prognostic indicator for a poor outcome in liver cirrhosis and HCC patients [34].

This study has some limitations, including the potential for sampling errors due to the very small size of the liver biopsy tissue specimen; inherent limitations of the study design for liver biopsy, such as variability, low accuracy, and risk factors for error; and the potential for selection bias owing to the single-center design. Sample size analysis and research power would be needed to validate the research findings. Further studies are also needed to determine whether the combination of sCD163 and other existing serum markers improve diagnostic performance.

## Conclusions

In conclusion, serum sCD163 levels reflect the progression of liver fibrosis and inflammation in patients with NAFLD and may be a potential biomarker of prognostic scrutiny. In cases where the serum sCD163 level is 800 ng/ml or higher, patients are predisposed to liver carcinogenesis and poor prognosis. Such patients with NAFLD and high serum sCD163 levels should, therefore, be subjected to NAFLD prognostic scrutiny and HCC screening.

## Electronic supplementary material

Below is the link to the electronic supplementary material.

**Figure S1**. Receiver-operating characteristic curve based on the serum sCD163 levels in patients with NAFLD. (a) fibrosis stage 3-4, (b) fibrosis stage 4, (c) Grade 2?3, (d) NAS ≧5. **Figure S2**. Comparison of sCD163 with other fibrosis markers (type 4 collagen 7S, hyaluronic acid, TIMP-1, P-III-P, WFA+M2BP). **Figure S3**. Changes in liver histology were examined in patients who underwent repeated biopsies (n=287) (5.7±3.1 years). **Figure S4**. Representative case of immunohistochemical staining, (a) CD68, (b) sCD163. **Table S1**. Receiver-operating characteristic curve based on the serum sCD163, type4collagen7S, hyaluronic acid, P-III-P, WFA+M2BP levels in fibrosis stage 3 or higher patients with NAFLD

## Data Availability

Data are available to researchers upon reasonable request from the corresponding author.

## List of abbreviations

AUC: Area under the curve
ELISA: Enzyme-linked immunosorbent assay
FFA: Free fatty acids
HCC: Hepatocellular carcinoma
hMDMs: Human monocyte-derived macrophages
IR: Insulin resistance
LPS: Lipopolysaccharide
NAFLD: Nonalcoholic fatty liver disease
NAS: NAFLD activity score
NASH: Nonalcoholic steatohepatitis
NASH CRN: NASH Clinical Research Network
NPV: Negative predictive value
PPV: Positive predictive value
ROC: Receiver operating characteristic
sCD163: Soluble CD163
BMI: Body mass index
ALT: Alanine aminotransferase
AST: Aspartate aminotransferase
γ-GTP: γ-glutamyltransferase
HOMA-IR: Homeostasis model assessment-insulin resistance level
P-III-P: Procollagen-III-peptide
TIMP-1: Tissue inhibitor of metalloproteinases-1
WFA^+^M2BP: Wisteria floribunda agglutinin Mac-2 binding protein
