# Supplementary material for: Soluble CD163 is a predictor of fibrosis and hepatocellular carcinoma development in nonalcoholic steatohepatitis
Source: BMC Gastroenterol. 2023 May 10;23:143. doi: 10.1186/s12876-023-02786-4 (PMC10173513; doi:10.1186/s12876-023-02786-4)
Supplement: Supplementary file 1 — Figure S1. Receiver-operating characteristic curve based on the serum sCD163 levels in patients with NAFLD. (a) fibrosis stage 3-4, (b) fibrosis stage 4, (c) Grade 2?3, (d) NAS ≧5. Figure S2. Comparison of sCD163 with other fibrosis markers (type 4 collagen 7S, hyaluronic acid, TIMP-1, P-III-P, WFA+M2BP). Figure S3. Changes in liver histology were examined in patients who underwent repeated biopsies (n=287) (5.7±3.1 years). Figure S4. Representative case of immunohistochemical staining, (a) CD68, (b) sCD163. Table S1. Receiver-operating characteristic curve based on the serum sCD163, type4collagen7S, hyaluronic acid, P-III-P, WFA+M2BP levels in fibrosis stage 3 or higher patients with NAFLD [file 12876_2023_2786_MOESM1_ESM.docx]

**Supplementary Materials**

**

**

**Figure S1.** Receiver-operating characteristic curve based on the serum sCD163 levels in patients with NAFLD

(a) fibrosis stage 3-4, (b) fibrosis stage 4, (c) Grade 2–3, (d) NAS ≧5

**

**

**Figure S2.** Comparison of sCD163 with other fibrosis markers (type 4 collagen 7S, hyaluronic acid, TIMP-1, P-Ⅲ-P, WFA+M2BP)

**
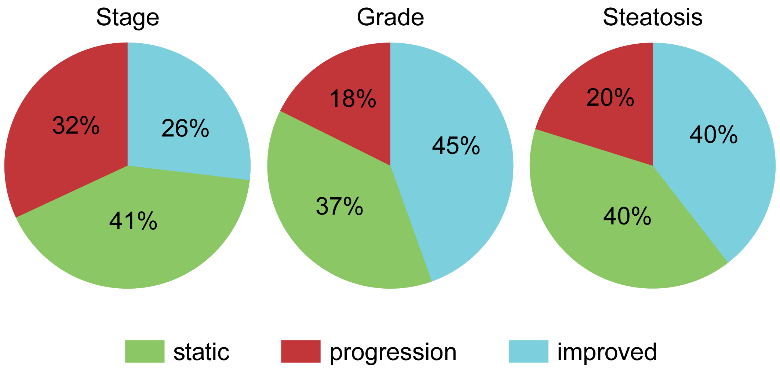
**

**Figure S3.** Changes in liver histology were examined in patients who underwent repeated biopsies (n=287) (5.7±3.1 years)

**
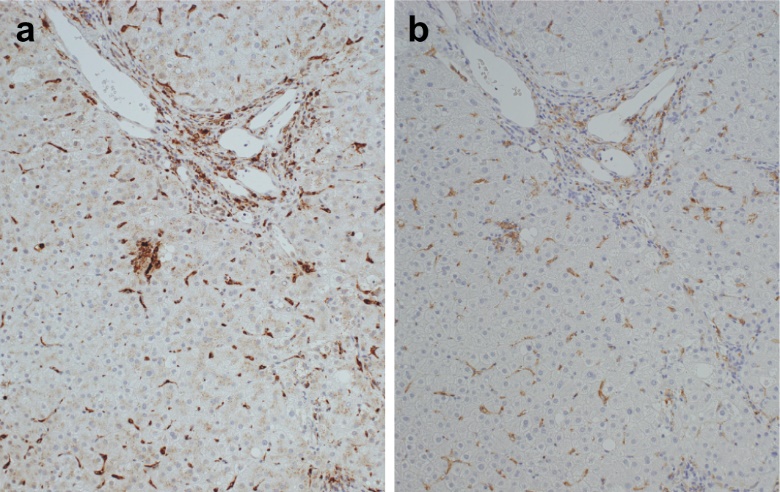
**

**Figure S4.** Representative case of immunohistochemical staining, (a) CD68, (b) sCD163

**Table S1.** Receiver-operating characteristic curve based on the serum sCD163, type4collagen7S, hyaluronic acid, P-III-P, WFA+M2BP levels in fibrosis stage 3 or higher patients with NAFLD

|  | AUC | 95%CI | cut off | sensitivity | specificity | PPV | NPV |
| --- | --- | --- | --- | --- | --- | --- | --- |
| s CD163 | 0.726 | 0.6447-0.7758 | 730 | 67 | 40 | 59.2 | 79 |
| Type4collagen7S | 0.7783 | 0.7155-0.8340 | 4.3 | 68.8 | 76.5 | 61.1 | 82 |
| Hyaronic acid | 0.7438 | 0.6763-0.8028 | 40 | 70.3 | 69.2 | 57.1 | 80 |
| P-Ⅲ-P | 0.6189 | 0.5485-0.6915 | 0.73 | 44.3 | 79.6 | 53.8 | 72.7 |
| WFA+M2BP | 0.7075 | 0.6354-0.7798 | 1.09 | 65.6 | 72.6 | 56.7 | 79.3 |
| TIMP-1 | 0.6755 | 0.5864-0.7631 | 172.5 | 71.1 | 60.5 | 41.6 | 84.1 |

P-III-P, procollagen-III-peptide; TIMP-1, Tissue inhibitor of metalloproteinases-1; WFA^+^M2BP, Wisteria floribunda agglutinin Mac-2 binding protein; sCD163, soluble CD163.
